# Use of IMMPACT domains in clinical trials of acupuncture for chronic pain: A methodological survey

**DOI:** 10.1371/journal.pone.0231444

**Published:** 2020-04-16

**Authors:** Lauren Giustti Mazzei, Cristiane de Cássia Bergamaschi, Marcus Tolentino Silva, Silvio Barberato Filho, Izabela Fulone, Mariana Del Grossi Moura, Caio Guimaraes, Luciane Cruz Lopes

**Affiliations:** Pharmaceutical Sciences Graduate Program, University of Sorocaba, Sorocaba, State of São Paulo, Brazil; University of Tasmania, AUSTRALIA

## Abstract

Acupuncture is one of the therapeutic resources used for the management of chronic pain. Variability in outcome measurements in randomized clinical trials of non-oncologic chronic pain (RCT-NOCP) generates inconsistencies in determining effects of treatments. The objective of this survey was to assess the adherence to the recommendations made by the Initiative on Methods, Measurement, and Pain Assessment in Clinical Trials (IMMPACT) in the measurement of RCT-NOCP of acupuncture. This methodological research made a systematic search for eligible studies from different sources of information. Eligible studies included those with number of patients ≥100, who randomized and allocated patients with chronic non-oncologic pain to be treated with acupuncture or with “sham” acupuncture, or non-acupuncture. This research included the recommendations for IMMPACT in the measurement of RCT-NOCP: presence of outcomes pain, physical function, emotional state and improvement perception of patient, the source of the outcome information pain and the tools used to measure such domains. From a total of 1,386 studies, 24 were included in this survey. Eleven studies presented low risk of bias. Pain outcome was measured in 23 studies, physical function in 22 studies, emotional state in 14 studies and improvement perception of patient in one study. As for the pain outcome, the patient was the information source in 50% of the studies. The measurement tools recommended for IMMPACT were included in eight studies (35%) that evaluated pain, one study that evaluated the emotional state (7%), and one study that evaluated the improvement perception and satisfaction of patient. It was observed that studies which did not adhere to the recommendations had more favorable results for acupuncture in the outcome pain. This study concludes that randomized clinical trials that used acupuncture to manage chronic pain failed to adhere to IMMPACT recommendations. Clinical societies and IMMPACT do not share the same recommendations. This fact reflects in the diversity of outcomes and instruments adopted in the studies, making it difficult to compare the results.

## Introduction

Pain is defined by the International Association for the Study of Pain as an unpleasant sensory and emotional experience associated with real or potential tissue damage [1]. Chronic pain is a complex symptom, with challenges for evaluation and management, both in clinical trials and in clinical practice [2]. It is estimated that 19% of the global population have chronic pain, and its management increases the development of new technologies and more effective treatments [3; 4].

Non-oncologic chronic pain (NOCP) is a public health problem, spending around 22% of primary health expenditures worldwide. The national costs in the United States are around $17.8 billion annually with pain medication. About 29% of American population takes five or more medications per day [5]. According to the STOP-PAIN Canadian project published in 2010, the average monthly cost of a person with chronic pain in health services waiting list for multidisciplinary pain treatment was $1,462 (CDN), and 95% of total expenses were paid by private financing [6].

A wide range of management strategies, both pharmacological and non-pharmacological, are available for chronic pain. The challenge is to understand the large published evidence for different treatments and define when and where to use them to get the best long-term results for the patient [3].

The guidebook published by the International Association for the Study of Pain for pain treatment in resource-poor conditions offers a tool to those who deal with people in economic poverty, living in developing countries and facing obstacles to acquire the necessary medicines for NOCP management. To control the chronic pain the guide suggests, besides drugs, the use of complementary resources such as acupuncture, therapeutic massages and other modalities, considering they have lower costs and less side effects [7]. Considering the prevalence of similar scenarios, the World Health Organization (WHO) has launched a Traditional Medicine strategy aiming to broaden the concepts of the competent authorities in regard to health’s improvement and patient’s autonomy [8].

The search for international guidelines that use acupuncture for the NOCP management in the adult population results in few findings and conflicting recommendations. Among the guidelines consulted, the recommendation to use acupuncture to several painful conditions is based on poor quality evidence due to variability in the methods of randomized clinical trials (RCT) [9–12]. In addition, these guidelines do not discriminate the power of recommendation, with the exception of Canada’s guidelines (evidence level "C": it recommends considering acupuncture in the chronic pain management) and Scotland (evidence level "A": it strongly recommends acupuncture for short-term pain relief in chronic low back pain and osteoarthritis) [10; 12].

Although considered the gold standard of primary studies for intervention evaluation, RCT with adequate randomization and blinding may not provide the best approach for developing a strong evidence base for pain management if its outcomes and assessment tools are not suitable [13]. These limitations have been recognized internationally, leading to the development of the Initiative on Methods, Measurement, and Pain Assessment in Clinical Trials (IMMPACT) in 2003.

This initiative brought together 27 experts from various universities, government agencies and the pharmaceutical industry in 2003 and generated a consensus, updated in 2008, with a core of four endpoint domains that should be considered in RCT’s for chronic pain [14], as well as their ideal instruments of measurement: pain intensity, evaluated by numerical scale of 0–10 or visual analogue scale; physical function, evaluated by the Multidimensional Inventory for Pain or Summary Inventory of Pain; emotional state, assessed by the Beck Depression Inventory or Mood Profile; and whole perception of patient improvement as assessed by the Global Impression of Patient Change questionnaire [15].

Besides the evaluation limitations and trial design, concerns were raised about how methods of analysis can outweigh clinically important positive outcomes, or overestimate the treatment effects. Multiple outcomes are often needed to properly evaluate the benefits of pain management, and sensitivity adjustments are required because if only the average of the results is considered, it may depict an ineffective treatment without taking into account the potential of effectiveness when the results are analyzed in subgroups [15].

The establishment of a standard set of outcome domains in RCT on chronic pain has several merits. It encourages researchers to consider chronic pain as a complex phenomenon that affects patients in multiple levels, protects against polarization of selective outcomes, which is a common problem throughout medical literature, and make it easier to perform systematic and meta-analyzes reviews, which allow researchers to provide estimates of the most accurate treatment effects by sharing common outcome data in individual trials [16].

This research checked out the adherence to IMMPACT recommendations in the measurement of RCT-NOCP of acupuncture. The verification of compliance with IMMPACT recommendations in RCT-NOCP allows greater transparency in decisions regarding the use of acupuncture as a viable alternative in this clinical condition.

## Methods

### Research design and question

This is a methodological inquiry study of RCT that used acupuncture for the treatment of NOCP. The PICO question of this study is: Did the RCT with NOCP patients treated with acupuncture compared to sham or non-acupuncture the measures results follow recommended by IMMPACT? The study protocol was published in the journal BMJ Open with identification number DOI: 10.1136/bmjopen-2016-014904 [17].

### Search sources

The systematic review by Vickers and partners selected 31 clinical trials obtained by means of a search in the bases chosen until December 2010. Additional research was carried out on the same bases, considering studies published until August 2017. Data sources included: Cumulative Index to Nursing and Allied Health Literature (CINAHL), Excerpt Medical Database (EMBASE), Medical Literature Analysis and Retrieval System Online (MEDLINE), Latin American and Caribbean Literature in Health Sciences (Lilacs), Allied and Complementary Medicine Database, Web of Science and Cochrane Central Registry of Controlled Trials, without language restrictions. The main terms "Chronic pain" and "Acupuncture" indexed in the Medical Subject Headings (MeSH) system were combined. First of all, it was searched for the isolated terms and their synonyms, and then was performed a second search, combining and crossing terms. The list of references or citations found in secondary studies has been checked to identify possibly eligible studies.

### Criteria and eligibility determination of studies

#### Outline

RCTs, whose number of patients was equal to or greater than 100. The date of recruitment of the first participant was not an eligibility criterion, differing from what has been stated in our protocol already published, to broaden the capture of studies, as described in detail previously [17].

#### Clinical condition

Patients aged 18 years or older with NOCP. The eligible pain conditions were: osteoarthritis, chronic or recurrent headaches, specific and nonspecific shoulder pains, and non-specific back or neck pain. For shoulder, back and neck pain, the pain episode should be at least four weeks. For osteoarthritis or headaches, the duration of pain was not necessary, since both are chronic in nature.

#### Intervention

Studies that included a group of patients treated with acupuncture, where acupuncture points or trigger points were stimulated with acupuncture needles and another group in which patients were treated with acupuncture simulated or without acupuncture and studies where blinding was adequate.

#### Exclusion criteria

Clinical trials with reports of neck or back pain associated with specific clinical conditions (eg. fractures resulting from osteoporosis).

Six reviewers, in pairs, independently assessed whether abstracts and titles met the eligibility criteria. Differences were resolved by consensus among all reviewers. To exclude duplicate articles, one reviewer analyzed all eligible articles and identified those who had one or more joint authors with the same title. In case of duplicate publication, we used the article with the most complete data.

### Data extraction

We adopted an Excel® spreadsheet for extracting data from all six reviewers independently. The reviewers were calibrated by extracting at least three articles and then performed consensus, in pairs and independently. The calibration process occurred until standardization of the extracted data.

Another reviewer confirmed the extraction to ensure the consistency of the answers obtained between the collaborators, arbitrating in the disagreements when necessary.

The data collected were: author, date of publication, country of origin, journal impact factor, recruitment date of the first participant, presence of outcome domains IMMPACT and tools used to measure the domains of outcomes, acupuncture method, patient clinical condition and duration of treatment. In addition, the study verified whether the outcomes of the pain outcome were reported by the patient (RRP), whether they were reported by clinicians (RRC), whether the result was reported by a third person (RRT) or a combination of the three.

The number of articles that measured pain, physical function, emotional state and perceived improvement and patient satisfaction were quantified according to the IMMPACT recommendations. We also computed the number of IMMPACT domains that were served in order to generate a score between 0–4 points. The score was described on average, standard deviation, median and interquartile range.

The journals impact factor review was carried out in the Journal Citation Reports database, and the year adopted for the consultation was 2016. The data extracted were subsequently submitted to statistical analysis.

### Risk of bias

A Cochrane’s tool modified version for bias risk assessment was used [18; 16]. The reviewers assessed the risk of bias for each clinical trial independently, regarding the following criteria: random sequence generation; allocation concealment; blinding of participants and personnel; blinding of outcome assessment; incomplete outcome data; selective reporting; and other biases.

The reviewers attributed as answer alternatives "definitely yes", "probably yes", "probably not" and "definitely not" for each of the domains [19]. Ultimately, "definitely yes" and "probably yes" were assigned as low risk of bias, while "definitely not" and "probably not" meant high risk of bias. The reviewers resolved disagreements through discussion, and a third person judged unresolved disagreements.

As described in the Cochrane Handbook for Systematic Reviews of Interventions, the bias risk assessment between studies can be judged to be low risk of bias when most studies are low risk in all seven domains and may be judged to be at high risk when the proportion of information from studies with high risk of bias is sufficient to affect the interpretation of results [16]. Review Manager Software version 5.3 was used to group bias-related risk data.

### IMMPACT definitions endpoint domains and recommended measurement instruments

The four recommended IMMPACT domains in 2008 are pain, physical function, emotional state and improvement perception and patient satisfaction, as defined:

1. Pain: includes several aspects of perception (eg. pain intensity, duration and frequency). The overall pain assessment examines how pain has changed during treatment. The recommended instruments for pain measurement are Visual Analog Scale or Numerical Rating Scale.
2. Physical function: refers to the participant's ability to perform daily activities (eg, tasks, walking, travel and self-care), strength and endurance. The recommended instruments for measuring physical function are the Multidimensional Pain Inventory or the Brief Pain Inventory interference scales.
3. Emotional status: refers to treatment associated with emotional distress (eg, depression, anxiety, anger, or irritability). The instruments recommended for measuring the patient's emotional state are the Beck Depression Inventory or the Profile of Mood States.
4. Patient's perception of improvement and satisfaction with treatment: refers to the participant's feeling about the treatment (if they feel that the positive characteristics of the treatment outweigh the negative ones). The recommended instrument for measuring patient improvement and treatment satisfaction is the Patient Global Impression of Change scale.

The correct applicability of the instrument was also measured (if the domain report was reported by the patient, clinician or by third parties).

### Statistical analysis

Year of publication, place of study, journal impact factor, and methodological quality assessment items (risk of bias) were evaluated descriptively.

The methodological survey protocol planned was the factors analysis associated with adherence to the IMMPACT domains by logistic regression, considering the domains of IMMPACT as dependent variables and the characteristics of the study as independent variables (year of publication, place of study, factor of periodic impact and items of methodological quality evaluation). Results were expressed as odds ratios with respective 95% confidence intervals (OR, 95% CI). All analysis were bilateral tests at a significance level of 0.05.

To explore the use of influence instruments recommended by IMMPACT on pain measurement, data from this outcome were pooled using meta-analysis. The standardized mean difference and 95% confidence interval were used as an effect measure. The random effects model proposed by DerSimonian and Laird was used [20]. The presence of heterogeneity was estimated by the calculation of I2.

The calculations were performed on STATA software version 14.2 and Review Manager version 5.3.

## Results

**Fig 1:**
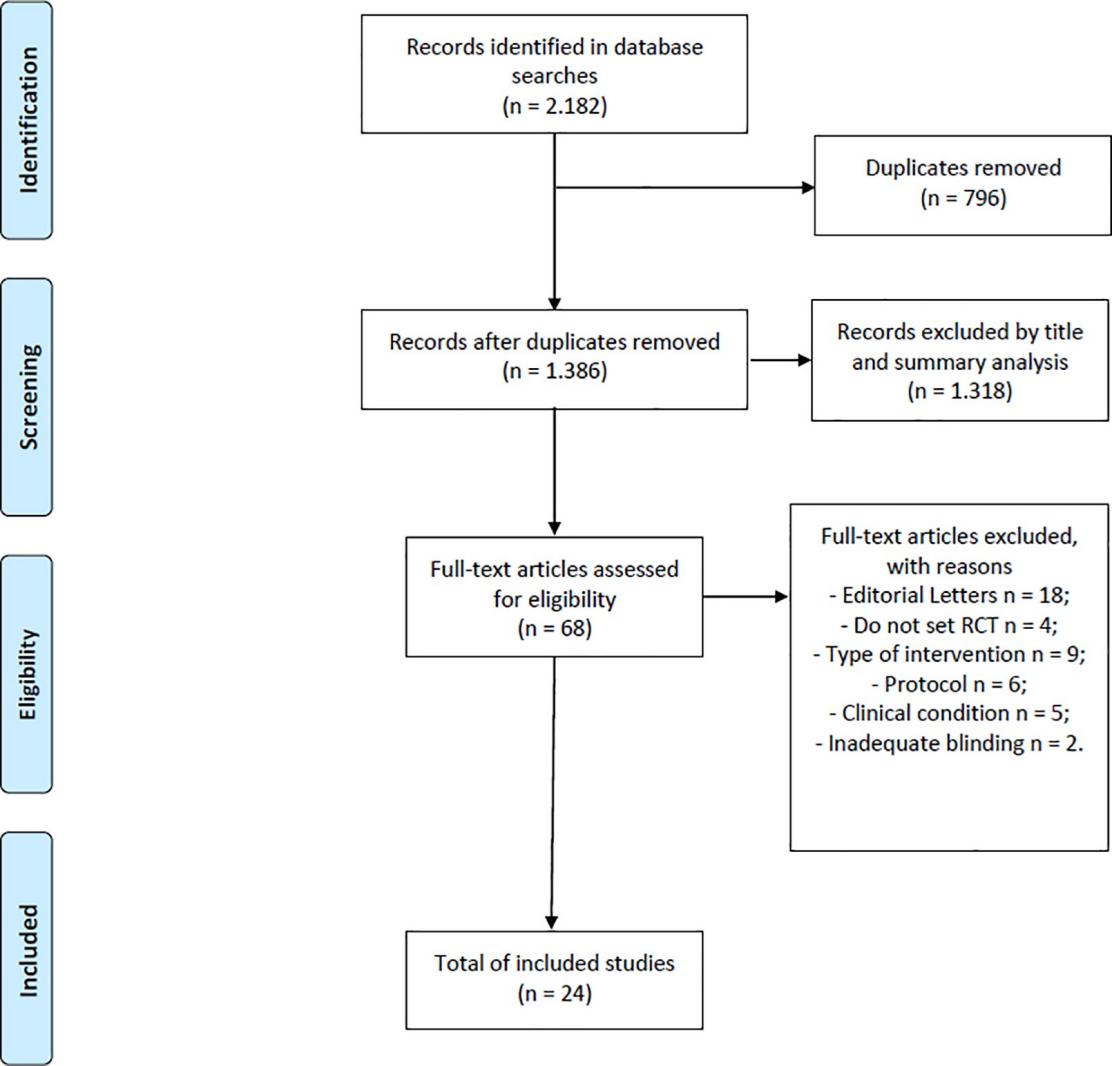
Flowchart of search and selection of studies.

There were 1.386 records, excluding duplicates. After selection of titles and abstracts, 1.318 records were excluded. Of the 68 articles that underwent full-text screening, 24 met the eligibility criteria (Fig 1).

### Studies characteristics

Table 1 presents the characteristics of the included studies. The studies were mostly published in Germany [21–35].

**Table 1:** Characteristics of included studies.

Variable	n (number of studies)
**Country of study**	
Germany	15
Australia	1
China	1
South Korea	1
United States	2
England	1
Italy	1
United Kingdom	2
**Impact factor, median (IQR)**	17,2 (4,21–20,78)
**Sponsor (n = 23)**	
Public agency	14
Private agency	9
**Protocol pre-registration**	12
**Sample size for analysis, median (IQR)**	231 (159,5–619,5)

IQR = interquartile range.

Studies from China, England, Australia, the United States and nine studies from Germany reported a source of public funding [21; 22; 25; 27; 29–31; 33; 35; 36–40]. Studies from the United Kingdom, Korea and six studies from Germany have declared private funding [23; 24; 26; 28; 32; 34; 41–43]; and one study, originating in Italy, did not state the source of funding [44].

From the 24 studies included, only half presented the protocol record number. The median impact factor of the journals in which the RCTs were published was 17.2 (4.21–20.78). The median sample size used for the primary analyzes in the trials was 231 (159.5–619.5).

The majority of patients included in these trials had chronic headache/migraine (n = 6) and osteoarthritis (n = 7). All the studies used traditional acupuncture, with the selection of points based on their energetic function for each clinical condition and considering the individual characteristics of the participants.

The type of control varied among the included studies: ten studies used sham acupuncture, with insertion of needles at sites that did not present acupuncture points [22; 25; 27–32; 39; 41]; seven studies used a non-penetrating needle, simulating the treatment session at the same points selected for the intervention group [21; 23; 24; 36; 37; 42; 44], or simulated electrostimulation, with a stimulator that emits audio and video signals, but with deactivated cables [43]; four studies used the waiting list [26; 34; 35; 38], and two studies used usual care [33; 40]. The details of each included study are presented in Table 2.

**Table 2:** Characteristics of included studies and outcomes measured.

Study	Clinical condition	Number of sessions / frequency / duration per treatment	Total sample size (n = 39,916)	Acupuncture group			Type of acupuncture	Control group			Type of control	Measured Outcomes	Scales
				n	Women	Average age		n	Women	Average age			
***Study that measured the four recommended domains***													
Chen 2013	Osteoarthritis	18 sessions/ 20 minutes	214	104	53	60.5	Traditional	109	57	60.4	Non-penetrating needle	Pain Physical function Emotional state Improvement of the patient	WOMAC SF-36 SF-36 PGIC
***Studies that measured three of the recommended domains***													
Liang 2011	Non-specific neck pain	9 sessions/ 3 weeks/ 30 minutes	178	88	63	36.7	Traditional	90	66	37.2	Acupuncture *sham*	Pain Physical function Emotional state	VAS NPQ SF-36
Jena 2008	Chronic headache	15 sessions/ 3 months	3,404	1,613	1,243	43.6	Traditional	1,569	1,219	43.7	Waiting list	Pain Physical function Emotional state	days SF-36 SF-36
Endres 2007	Chronic headache	10 sessions/ 5 weeks/ 30 minutes	409	209	163	39.2	Traditional	200	158	38.9	Non-penetrating needle	Pain Physical function Emotional state	GCPS SF-12 SF-12
Haake 2007	Non-specific back pain	10 sessions/ 5 weeks/ 30 minutes	1,162	387	222	49.6	Traditional	387	247	49.2	Non-penetrating needle	Pain Physical function Emotional state	GCPS SF-12 SF-12
Diener 2006	Migraine	10 sessions/ 6 weeks/ 30 minutes	960	290	247	37.1	Traditional	317	257	38.3	Acupuncture *sham*	Pain Physical function Emotional state	GCPS SF-12 SF-12
Scharf 2006	Osteoarthritis	10 sessions/ 6 weeks/ 20–30 minutes	1,007	330	220	62.8	Traditional	367	255	63.0	Acupuncture *sham*	Pain Physical function Emotional state	WOMAC SF-12 SF-12
Brinkhaus 2006	Non-specific back pain	12 sessions/ 8 weeks/ 30 minutes	298	147	93	59.1	Traditional	75	55	58.2	Non-penetrating needle	Pain Physical function Emotional state	VAS PDI ADS
Witt_a 2006	Osteoarthritis	15 sessions/ 3 months	3,553	322	182	60.6	Traditional	310	198	61.9	Usual care	Pain Physical function Emotional state	WOMAC WOMAC SF-36
Linde 2005	Chronic headache	12 sessions/ 8 weeks/ 30 minutes	302	145	129	43.3	Traditional	81	73	41.3	Acupuncture *sham*	Pain Physical function Emotional state	Questionário PDI ADS
Melchart 2005	Chronic headache	12 sessions/ 6 weeks/ 30 minutes	270	132	95	42.3	Traditional	65	46	43.4	Acupuncture *sham*	Pain Physical function Emotional state	PDI SF-36 ADS
Witt 2005	Osteoarthritis	12 sessions/ 8 weeks/ 30 minutes	294	149	105	64.5	Traditional	75	49	63.4	Acupuncture *sham*	Pain Physical function Emotional state	WOMAC SF-36 ADS
White 2004	Non-specific neck pain	8 sessions / 4 weeks/ 20 minutes	135	70	46	53.9	Not informed	65	41	52.8	Simulated electrical stimulation	Pain Physical function Emotional state	VAS NDI SF-36
***Studies that measured two of the recommended domains***													
Irnich 2001	Non-specific neck pain	5 sessions/ 10 weeks/ 30 minutes	177	56	39	52.3	Traditional	61	40	52.2	Acupuncture *sham*	Pain Physical function	VAS SF-36
Hinman 2014	Osteoarthritis	12 sessions/ 12 weeks/ 20 minutes	282	70	32	64.3	Traditional	71	40	62.7	Waiting list	Pain Physical function	VAS WOMAC
Cho 2013	Nonspecific back pain	12 sessions/ 6 weeks	116	57	47	42.3	Traditional	59	51	41.7	Acupuncture *sham*	Pain Emotional state	VAS BDI
Molsberger 2011	Non-specific shoulder pain	15 sessions/ 20 minutes	424	154	88	50.3	Traditional	135	89	51.3	Acupuncture *sham*	Pain Physical function	VAS Teste físico
Foster 2007	Osteoarthritis	6 sessions/ 6 weeks/ 30 minutes	352	117	71	63.1	Traditional	119	66	62.8	Non-penetrating needle	Pain Physical function	WOMAC WOMAC
Thomas 2006	Nonspecific back pain	10 sessions/ 30 minutes	239	160	99	42.0	Traditional	81	46	44.0	Usual care	Pain Physical function	McGill OPI
Witt_b 2006	Non-specific neck pain	15 sessions	13,846	1,753	1,225	49.8	Traditional	1,698	1,152	51.4	Waiting list	Pain Physical function	Wheeler HFAQ
Witt_c 2006	Low back pain	10 sessions/ 3 months	11,378	1.451	837	53.1	Traditional	1,390	791	52.6	Non Acupuncture	Pain Physical function	LBPRS HFAQ
Berman 2004	Osteoarthritis	23 sessions/ 26 weeks/ 20 minutes	570	190	120	65.2	Traditional	191	118	66.2	Non-penetrating needle	Pain Physical function	WOMAC HFAQ
***Studies that measured one of the recommended domains***													
Facco 2008	Migraine	20 sessions/ 10 weeks	160	40	18	35.2	Traditional	40	16	39.4	Non-penetrating needle	Physical function	MIDAS
Molsberger 2002	Low back pain	12 sessions/ 4 weeks/ 30 minutes	186	65	29	49.0	Traditional	61	28	50.0	Acupuncture *sham*	Pain	VAS

WOMAC = Western Ontario and McMaster Universities Osteoarthritis Index, SF-36/SF-12 = Short—Form Health Survey, PGIC = Patient Global Impression of Change, VAS = Visual Analogue Scale, NPQ = Neuropathic Pain Questionnaire, GCPS = Von Korff Chronic Pain Grade Scale, PDI = Pain Disability Index, ADS = Allgemeine Depressionsskala, NDI = Neck Disability Index, BDI = Beck Depression Inventory, OPI = Owestry Pain Index, LBPRS = Low Back Pain Rating Scale, HFAQ = Hannover Functional Ability Questionnaire, MIDAS = Migraine Disability Assessment Scale.

**Fig 2:**
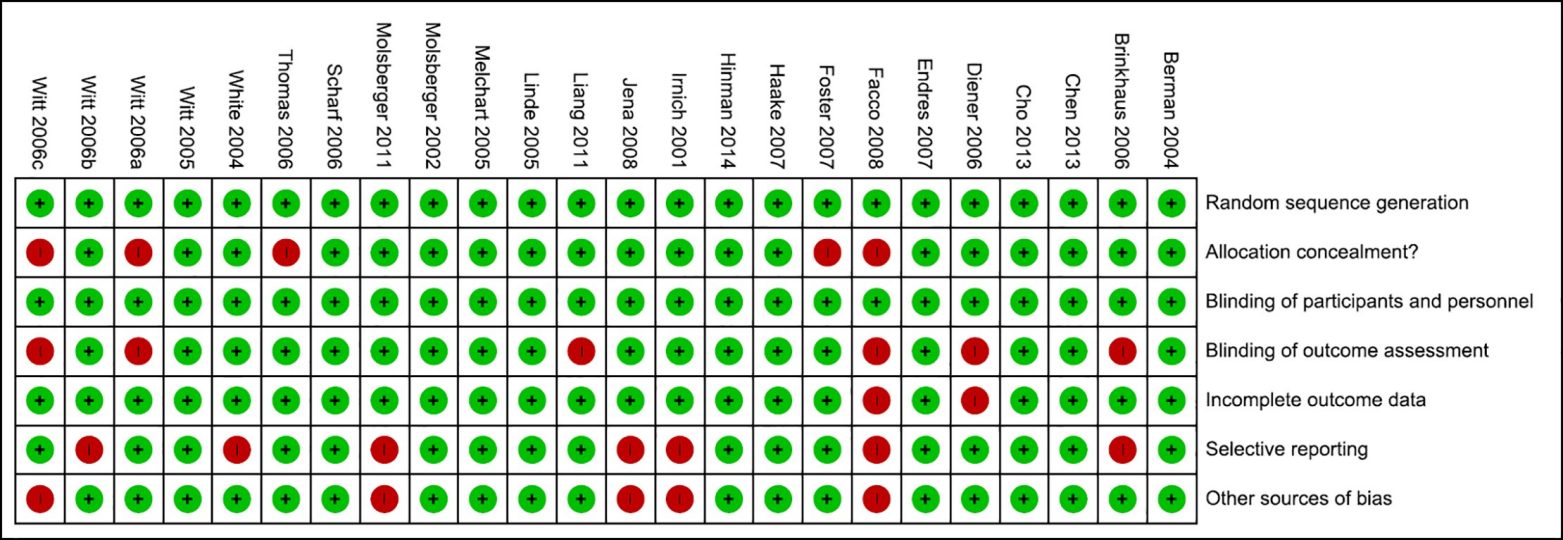
Summary of bias risk of individual studies.

The clinical trials included presented, in the general context, low risk of bias (Fig 2). In the criterion of allocation secrecy, a study presented high risk, since the allocation to the clinician occurred according to the availability of commitments and convenience for the patients [40], and four studies did not present the criterion with clarity [33; 35; 42; 44].

In six studies the authors did not clarify the blinding (or lack thereof) of outcome assessors [21; 22; 33; 35; 39; 44]. In one study there was no clarity as to whether outcomes were adequately reported due to the absence of protocol record in their trial [44], and a study for presenting incomplete outcome when compared to the methods reported in their protocol [22].

Seven studies presented high risk in the criterion of selective outcome [21; 25; 26; 29; 34; 43; 44]. In 5 studies, there was not found sufficient information for other sources of bias [25; 26; 29; 35; 44].

### Outcomes domains recommended by IMMPACT and instruments used in included studies

The most commonly reported outcomes were pain and physical function. The participant's emotional state was assessed in 14 of the 24 studies, and the perception of improvement and patient satisfaction was addressed in only one study [37]. Of the included studies, 22 were published as of 2004, none referred to the IMMPACT recommendations published in 2003 and updated in 2008 (Table 3).

**Table 3:** Domains recommended by IMMPACT, instruments used in the included studies and source of information on the pain outcome.

Outcome domains (n)	Evaluation scales	Number of studies (n)
Pain (23)	VAS	8
	WOMAC	6
	GCPS	3
	Others	6
Physical function (22)	WOMAC	4
	SF-36	5
	SF-12	4
	others	9
Emotional state (14)	BDI	1
	ADS	4
	SF-36	5
	SF-12	4
Perception of improvement and patient satisfaction (1)	PGIC	1
Reference to IMMPACT recommendations* (22)		0
Source of pain information reported by (n = 23):		
Patient		12
Clinical		1
Patient and clinical		10

VAS = Visual Analogue Scale, WOMAC = Western Ontario and McMaster Universities Osteoarthritis Index, GCPS = Graded Chronic Pain Scale, BDI = Beck Depression Inventory, ADS = Allgemeine Depressionsskala, SF = Short Form, PGIC = Patient Global Impression of Change.

*RCT published since 2004.

The endpoint pain was reported only by the patients in 12 of the 24 included studies (Table 3) [21; 23; 24; 27; 28; 31; 32; 35; 39–41; 43].

In ten studies the outcome was reported by both, clinical and patient (43.4%) [22; 25; 26; 29; 33; 34; 36–38; 42]. Only in one study were the results reported only by clinicians [30]. The source of the information was only described for the pain outcome, as the studies did not describe it for the other outcomes.

#### Pain

Pain was the major outcome in 23 RCTs. In eight studies, the pain assessment instrument used was one of those recommended by IMMPACT, the Visual Analogue Scale [21; 25; 29; 30; 38; 39; 41; 43]. Three studies used as an evaluation instrument the Von Korff Chronic Pain Grade Scale (GCPS) [22–24]. In six studies, Western Ontario and McMaster University Osteoarthritis Index (WOMAC) was the instrument used [31; 32; 34; 36; 37; 42]. A pain questionnaire that encompasses its intensity and number of days was adopted in two studies [26; 27]. Thomas et al. [40] adopted in their study the McGill pain questionnaire, while Witt et al. [33] used the Low Back Pain Rating Scale (LBPRS), Melchart et al. [28] used the German version of the Pain Disability Index (PDI), and Witt et al. [35] used the Wheeler scale.

#### Physical function

None of the studies evaluating physical function used the instruments recommended by IMMPACT. The physical function was an outcome evaluated in 22 studies. Four studies used the Short-Form Health Survey-12 [22–24; 31]. The Short-Form Health Survey-36 was the instrument adopted in five studies [25; 26; 28; 32; 37]. The Pain Disability Index (PDI) was used in studies by Linde et al. (2005) and Brinkhaus et al. [21], and the Western Ontario and McMaster Universities Osteoarthritis Index (WOMAC) was used in four studies [34; 36; 38; 42]. Thomas et al. [40] used as an instrument of evaluation of physical function the Owestry Pain Index; White et al. [43] used the Neck Disability Index; Facco et al. [44] used the Migraine Disability Assessment Test (MIDAS); Liang et al. [39] used the Neuropathic Pain Questionnaire (NPQ) and Molsberger et al. [29] adopted physical tests to evaluate this function. The Hannover Functional Ability Questionnaire was adopted in two studies [33; 35].

#### Emotional state

The patient’s emotional state was an outcome evaluated in 14 RCTs. The Short-Form Health Survey 12 and 36 were the instruments used in nine studies [22–24; 26; 31; 34; 37; 39; 43].

Four studies used the Allgemeine Depressionsskala [21; 27; 28; 32]. The Beck Depression Inventory, recommended by IMMPACT, was used as instrument to measure the emotional state of patients only in the study by Cho et al [41].

#### Improvement perception and patient satisfaction

The outcome perception of improvement and patient satisfaction with the treatment offered was evaluated only in a study conducted by Chen et al. [37], which used as evaluation instrument the Patient Global Impression of Change, recommended by IMMPACT.

### Associated factors

No association was found between the publication’s year of studies, published until 2006 and from 2007 (OR = 0.75; IC 95%: 0.15–3.83; p = 0.72); the corresponding author’s country, whether or not belonging to the European continent (OR = 2.06; IC 95%: 0.28–15.36; p = 0.48), or of German origin (OR = 4.0; IC 95%: 0.69–23.09; p = 0.12); the impact of the publication period above 7 (OR = 1.0; IC 95%: 0.94–1.06; p = 0.88); the methodological quality, considering studies that met 6 or 7 critical evaluation criteria (OR = 1.4; IC 95%: 0.28–7.02; p = 0.862); and the publication of the study protocol OR = 1.04; IC 95%: 0.32–3.10; p = 0.68) (Table 4).

**Table 4:** Factors associated with adherence to the domains recommended by IMMPACT.

Variables explored	Studies n (%)	OR	CI 95%	p value
**Year of publication**				
2001–2006	14 (58)	1.00		
2007–2014	10 (42)	0.75	(0.15–3.83)	0.72
**Corresponding author's country**				
Non-european	5 (21)	1.00		
European	19 (79)	2.06	(0.28–15.36)	0.48
Non-german	9 (38)	1.00		
German	15 (62)	4.00	(0.69–23.09)	0.12
**Periodic impact fator**				
≤7	11 (46)	1.00		
>7	13 (54)	1.00	(0.94–1.06)	0.88
**Methodological quality (bias)**				
≤ 5	8 (44)	1.00		
> 5	16 (66)	1.40	(0.28–7.02)	0.86
**Protocol**				
Non protocol	12 (50)	1.00		
With protocol	12 (50)	1.04	(0.32–3.10)	0.68

OR = *odds ratio*, CI = confidence interval.

### Secondary analysis

**Fig 3:**
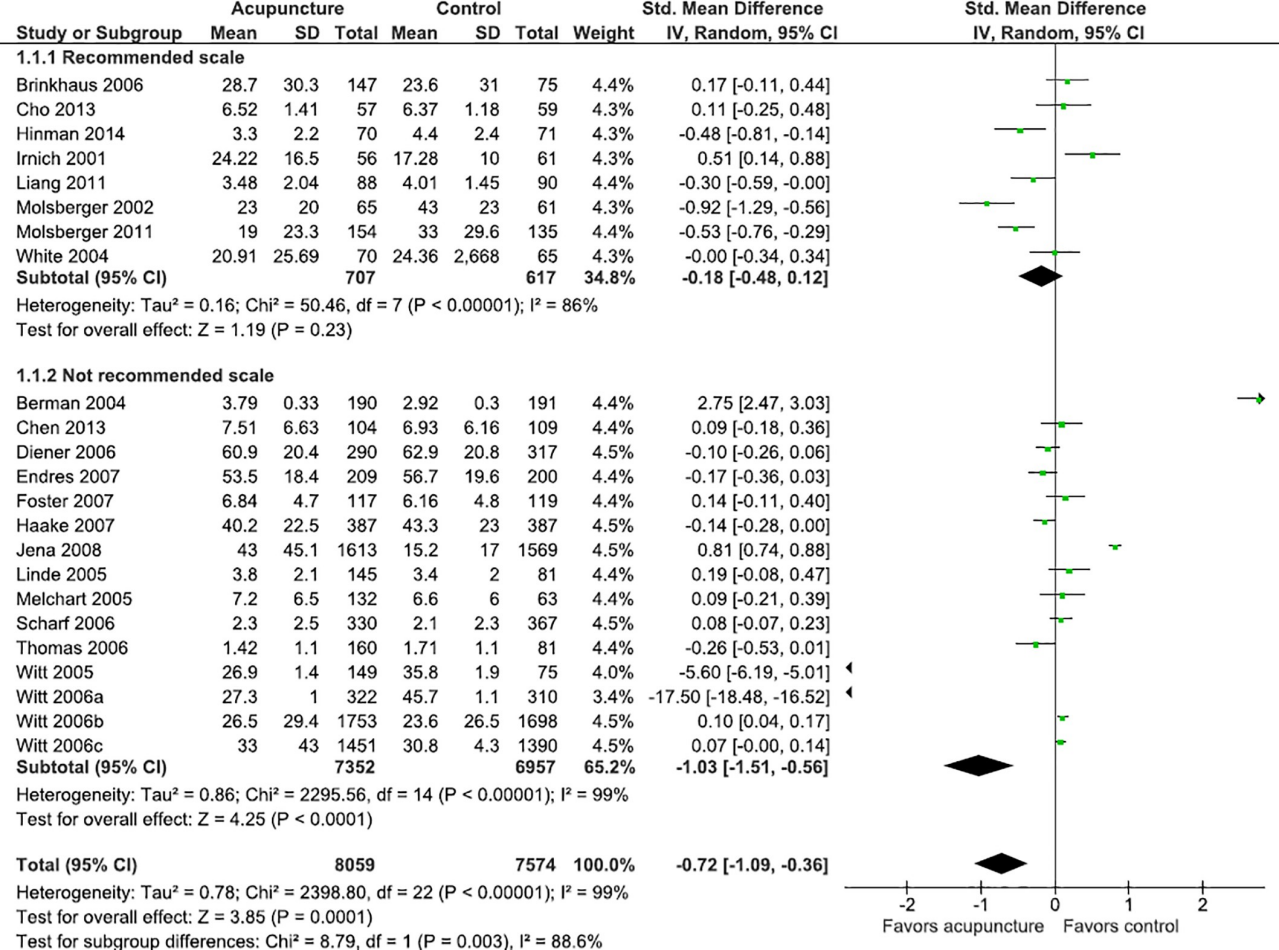
Meta-analysis of the effect of acupuncture on pain stratified by the use of recommended scale or not.

Studies that did not adhere to the IMMPACT recommendations had favorable results for acupuncture in the pain outcome (MD = -1.03; IC 95%: -1.51; -0.56; I^2^ = 99%), while those who followed the recommendations did not have statistically significant differences (MD = -0.18; IC 95%: -0.48; 0.12; I^2^ = 86%) (Fig 3). To investigate this finding, subgroup analysis was performed considering the clinical condition (back pain, neck pain, osteoarthritis and low back pain), and stratified by the use of recommended scales for the measurement of pain. Studies in which the clinical condition was headache or migraine were not analyzed because none of these studies used a recommended instrument, and the shoulder pain condition was addressed in only one study.

**Fig 4:**
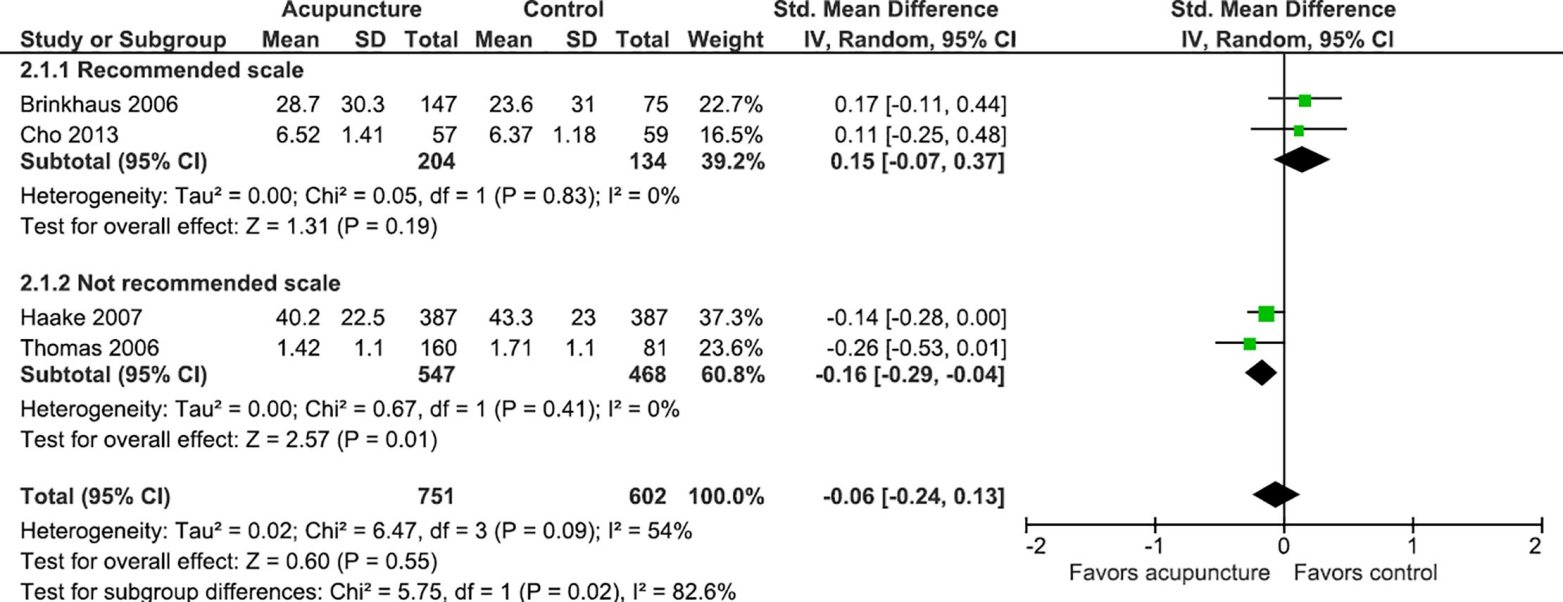
Meta-analysis of the effect of acupuncture in patients with back pain stratified by the use of recommended scales or not.

**Fig 5:**
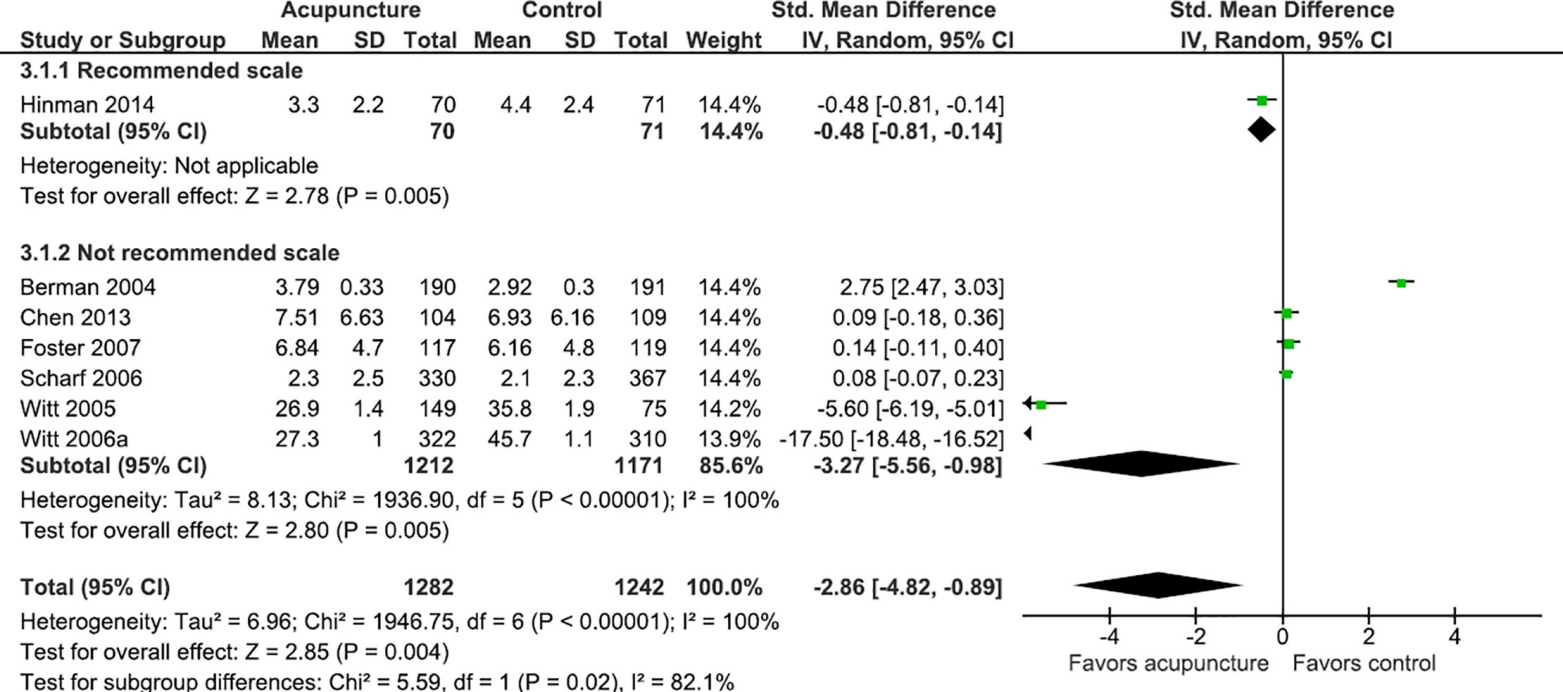
Meta-analysis of the effect of acupuncture in patients with osteoarthritis stratified by the use of recommended scales or not.

**Fig 6:**
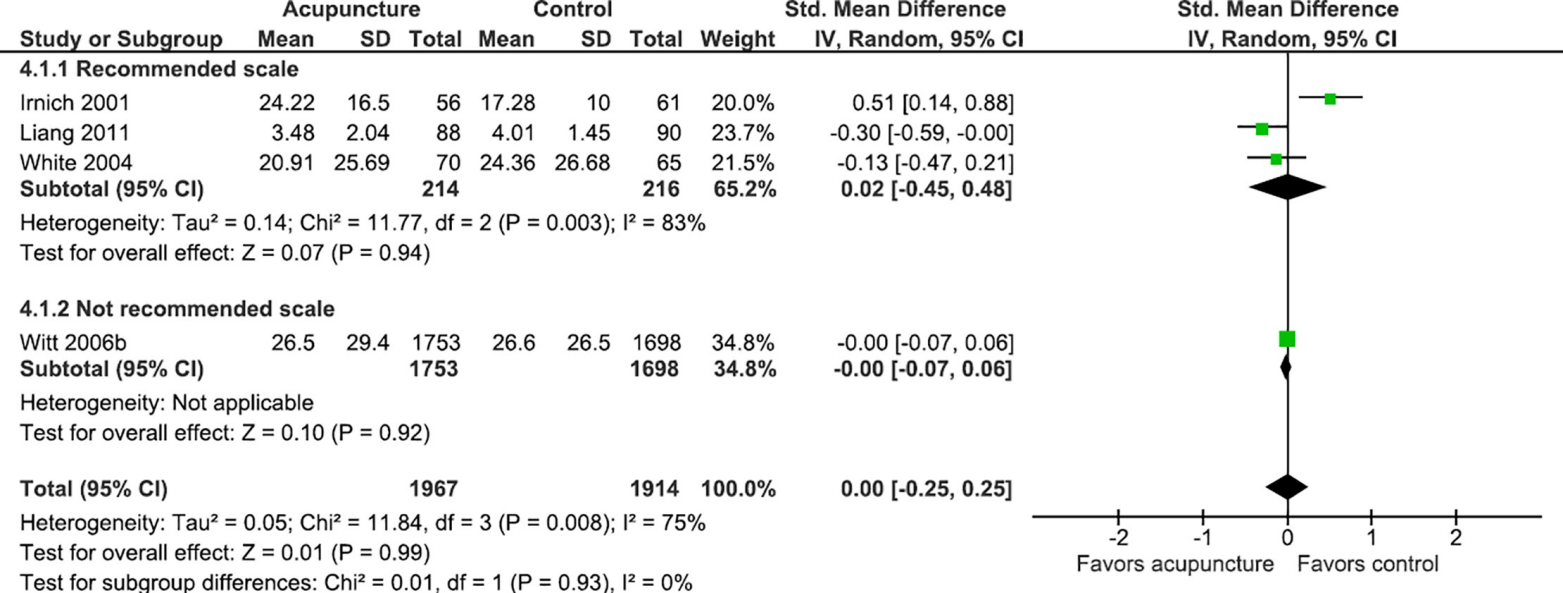
Meta-analysis of the effect of acupuncture in patients with low back pain stratified by the use of recommended scales or not.

**Fig 7:**
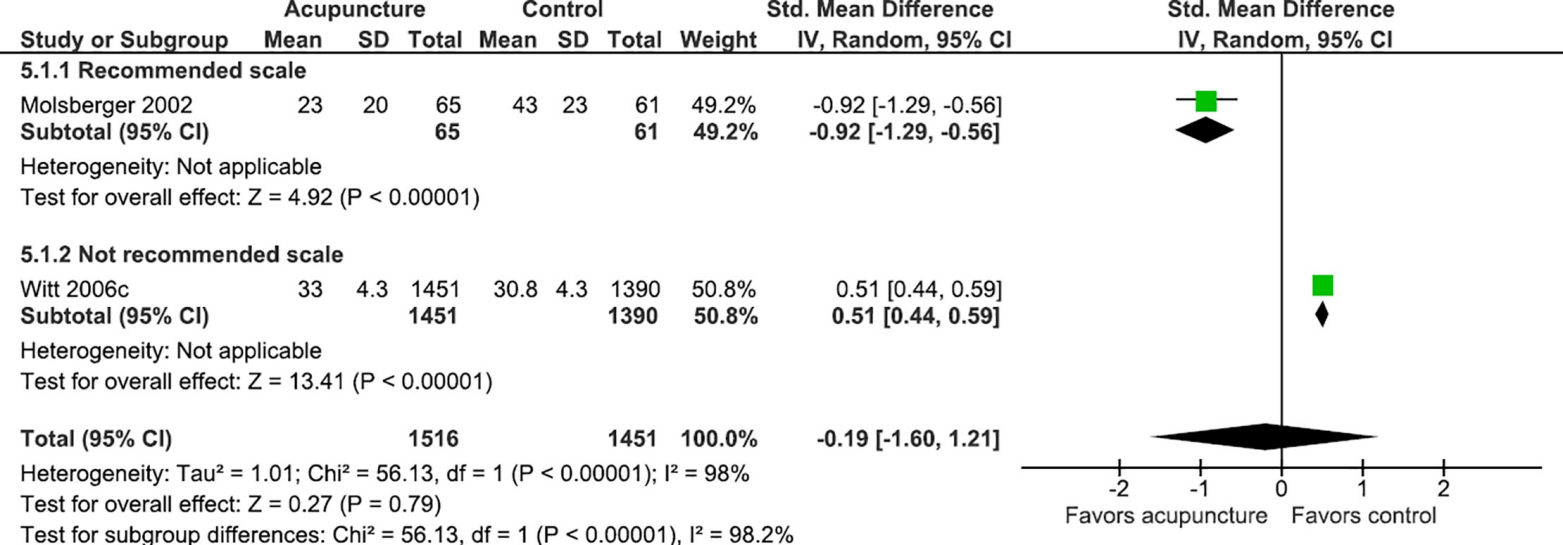
Meta-analysis of the effect of acupuncture in patients with neck pain stratified by the use of recommended scales or not.

In studies where the clinical condition was back pain and osteoarthritis, the data suggests that the use of non-recommended scales may overestimate the effect of acupuncture on gauging pain, and in studies where the clinical condition was low back pain, the use of scales may underestimate the effect of acupuncture on pain (Figs 4–7).

## Discussion

### Summarizing of results

In this survey, it was observed that most of the RCT were published in Germany and developed with public funding. The sample size of the arms analyzed in the studies ranged from 40 to 1,753 patients, and the most prevalent clinical conditions being chronic headache or migraine and osteoarthritis treated exclusively with traditional acupuncture.

None of the studies published since 2004 explicitly mentioned the IMMPACT recommendations, though they met at least two of the four areas recommended by the initiative and only one study assessed the four domains. The most reported outcomes in the studies were pain and physical function.

For evaluation of pain outcome, one-third of the studies used one of the recommended instruments. It is important to note that only for this outcome, the clinical trials described the source of the information; it was mostly reported by patients or by clinicians and patients.

No study evaluating physical function used the recommended instruments, and for the emotional state outcome, only one study used a recommended instrument. The only study that evaluated the perception of improvement and patient satisfaction with the treatment used the scale recommended by IMMPACT.

Variables such as year of study publication, country of origin, impact factor of the journal, absence of risk of bias and previous publication of protocol did not present a statistically significant influence to adherence to the outcome domains recommended by IMMPACT.

The use of instruments not recommended by the Initiative seems to overestimate the acupuncture effect on the pain of the patients with back pain and osteoarthritis when compared with studies using a recommended instrument.

### Relation with existing literature

The aim of IMMPACT recommendations was to provide baseline data to assess and compare the impact of treatments on symptoms, function, well-being and quality of life in the general health of patients with chronic pain [15].

The importance of considering these domains of outcomes is observed in a qualitative study that evaluated the patients' expectations for the outcomes of treatments based on complementary and alternative medicine. The authors pointed out that patients expect the proposed treatments to have an impact on pain, improvement of function and general well-being [45].

The low adherence to the recommendations is evidenced when the other data of this study were analyzed. After fifteen years of the first recommendation published by the Initiative, this study points out that among the 24 selected studies that used acupuncture as treatment for NOCP, only one of the four recommended outcome domains was addressed, and only 50% of them evaluated three outcomes among those recommended, pain and physical function being the most evaluated outcomes.

Failure to consider important outcomes to the patient may limit the validity and usefulness of clinical trials [46].

As far as sources of information are concerned, the results reported by the patient are particularly important for conditions involving symptoms such as pain and fatigue, where objective measures of patient perceptions are not available. Results reported by clinicians can be judged and underestimated [47]. In this survey, the endpoint pain was reported exclusively by the patients in 50% of the 24 included studies.

It was also possible to identify that the measurement instruments used were validated, but only eight studies used a recommended instrument when measuring pain and one study used a recommended instrument to assess the emotional state.

Although this survey has stratified studies by their used scales, analyzed the subgroups by clinical condition and explored associated variables, the results are statistically insignificant and very heterogeneous. Additionally, the use of scales not recommended in the measurement of the pain endorsement overestimates the effect of acupuncture when compared to studies using a recommended instrument under certain clinical conditions.

It is clear that the use of different scales in the measurement of the domains explored does not contribute to the clinical decision-making for or against the use of acupuncture in the control of NOCP.

A methodological survey explored adherence to IMMPACT recommendations in clinical trials that used opioids in the treatment of NOCP. The authors pointed out that more recent studies originating in North America and published in journals with high impact factor were more likely to adhere to the areas recommended by the Initiative. After 12 years of the first recommendation, the authors observed that there was no increase in the report of the recommended domains in temporal progression [48].

This propensity can also be observed in this survey, with a trend towards adherence to the areas recommended by studies from European countries, with low risk of bias and previous protocol publication, but these variables did not present significant results in the statistical analysis.

A systematic review with a meta-analysis of individual data from 17,992 patients with chronic pain pointed out that acupuncture is superior to placebo, with modest differences. The protocol of this review was well delineated, with eligibility criteria that sought to select good studies for extraction, such as those adopted in this study, but points as a possible limitation the multiple outcomes combination, measured at different times and with different instruments [49].

This limitation is reinforced in a review which points out that growing evidence supports the acupuncture use for chronic pain in many conditions as adjuvant in reducing the use of analgesic drugs. The authors emphasize as limitations the heterogeneity of the studies, not only in interventions or general characteristics of the population, but also the outcomes measured [50].

In international recommendations for specific clinical conditions, the principal instrument of evaluation indicated in cases of osteoarthritis recommended by the International Knee Documentation Committee is WOMAC; for chronic headache, the scale recommended by the International Headache Society is VAS; for neck pain, the Orthopedic Section of the American Physical Therapy Association recommends the use of NDI or the Patient-Specific Functional Scale; for back and lower back pain, the scales recommended by The American College of Physicians are VAS, ODI and Roland Morris Disability Questionnaire [51–54].

IMMPACT does not discriminate the use of other specific and validated instruments to measure the recommended outcomes, but emphasizes that the use of the suggested instruments facilitates the systematic reviews conception and meta-analysis allowing more precise estimates of effects, favors the sharing of common results data, and protects RCT against bias from selective outcomes [15].

Initiative researchers, along with the Outcome Measures in Rheumatology group, support the adoption of recommendations in designing chronic pain RCT, noting that the use of a standard of valid measurement domains and instruments can accelerate the development of treatments and facilitate the comparison between studies, producing more robust results [55].

With the increase in the number of RCT-NOCP, attention and research on the methodological aspects adopted in the studies should increase, with the potential to improve the sensitivity of the trial, and, finally, to provide more significant evaluations of the treatments for this condition.

### Limitations and strengths

The methods and sample size of the included studies in this research make it an important exploratory study, since it considered eligible high quality studies and a sample with a number greater than 100, with adequate blinding. The information sources consulted for systematic search did not include Asian bases and may represent possible losses in the studies capture.

The analysis adopted in this study did not identify factors associated with adherence to the IMMPACT recommendations. It is possible that other statistical methods might be used to extend the variables exploration and then identify factors that justify associative tendencies.

### Clinical implications

The adoption of important outcomes in disease impact assessing on the patient's life and the standardization of instruments can ease the conception of results that are close to the truth, substantiated by more robust analysis.

There is a real need for a dialogue between clinical societies and IMMPACT in order to increase the strength of therapeutic recommendations in the NOCP treatment in different clinical conditions.

## Conclusion

Studies that used acupuncture in the CNCD treatment did not adhere to the IMMPACT recommendations, which may favor the acupuncture performance in the pain outcome.

## Supporting information

S1 Checklist (DOC)
